# Effect of Buffalo Breed on the Detailed Milk Composition in Guangxi, China

**DOI:** 10.3390/foods12081603

**Published:** 2023-04-10

**Authors:** Mahmoud Abdel-Hamid, Li Huang, Zizhen Huang, Ehab Romeih, Pan Yang, Qingkun Zeng, Ling Li

**Affiliations:** 1 Guangxi Key Laboratory of Buffalo Genetics, Reproduction and Breeding, Guangxi Buffalo Research Institute, Chinese Academy of Agricultural Sciences, Nanning 530001, China; 2 Dairy Science Department, Faculty of Agriculture, Cairo University, Giza 12613, Egypt; 3 Key Laboratory of Buffalo Genetics, Breeding and Reproduction Technology, Ministry of Agriculture and Rural Affairs, Nanning 530001, China; 4 College of Animal Science and Technology, Guangxi University, Nanning 530004, China

**Keywords:** buffalo milk, Murrah, Nili-Ravi, Mediterranean, chemical composition

## Abstract

Buffalo is the second source of milk in the world, and its milk is rich in nutritive components. It is well-known that breed influences milk composition. This work aimed to compare the detailed milk composition of three buffalo breeds (Murrah, Nili-Ravi, and Mediterranean) housed under the same environmental conditions. Mediterranean buffalo milk showed a significantly higher content of fat, protein, and some fatty acids. Moreover, the milk from the Mediterranean breed was characterized by the highest content of sphingomyelin (SM), cholesterol, and lanosterol. However, the Murrah buffalo milk contained the highest amount of total unsaturated fatty acids, phosphatidylinositol, and whey proteins. Furthermore, the Nili-Ravi buffalo milk was characterized by the highest content of total saturated fatty acids, phosphatidylglycerol, squalene, lathosterol, stigmasterol, beta-sitosterol, and casein fractions. Nevertheless, the lactose and amino acid profiles of the milk remained almost similar across the three buffalo breeds. The generated results in this study enable a comprehensive understanding of the milk constituent variability that is linked to buffalo breeds, which may support the acquirement of essential scientific knowledge on milk ingredient–processing interactions that will offer a foundation of knowledge for Chinese dairy processors in terms of milk processability and innovation.

## 1. Introduction

Buffalo (*Bubalus bubalis*) is one of the most significant cattle for milk production worldwide. In 2020, buffalo milk production represented 15.27% (139 million tons) of the world’s total milk production; moreover, its annual production growth rate was reported to be 4.5% in 2020 [1]. Buffalo milk has grown in popularity because its mild flavor and high content of protein, fat, vitamins, and other nutrients compared with cow milk [2].

Buffaloes are generally distributed in Asia, the Middle East, and Europe [3]. China was the third country after India and Pakistan in dairy buffalo breeding in the world in 2020. The total milk production in China reached 39.65 million tons in 2022 (USDA report 6/2022), and dairy buffalos in China produced a total of around three million tons of milk. In China, buffalo milk is mostly produced and consumed in southwestern provinces, including Guangxi, Yunnan, and Guizhou.

The variation between buffalo milk and cow milk has been widely reported in previous studies [4]. In this context, a higher gross chemical composition (total solids, fat, protein, and lactose) was reported in buffalo milk than in cow milk [2,5]. Moreover, the fat globule size was larger in buffalo milk than in cow milk [6,7]. Furthermore, buffalo milk is rich in minerals and vitamins compared with cow milk [8]. 

Buffalo milk properties fluctuate due to a range of interconnected factors, including the breed, season, feeding, animal health, and the stage of lactation. Furthermore, changes appear in milk composition between breeds and individuals within a breed, which is partly due to genetic differences [9]. The water buffalo consists of two subspecies, namely, the swamp buffalo and the river buffalo [10,11]. River buffalo are mostly reared for their milk. Murrah, Nili-Ravi, and the Mediterranean are highly distributed river buffalo breeds due to their efficiency in milk production [12]. 

The detailed chemical composition of milk from various buffalo breeds in China has not been well-studied. In this context, it has been reported that milk from the Murrah and Nili-Ravi buffalo breeds showed no significant differences in the percentages of the total solid, protein, fat, fatty acid, amino acid, and protein sub-fractions [13,14]. Moreover, Zhou [15] analyzed the gross milk composition and amino acid content of milk from the Murrah and Nili-Ravi buffalo breeds and demonstrated that Murrah milk contained higher protein and total solids than Nili-Ravi buffalo milk. Regarding amino acids, milk from the Murrah breed contained significantly higher concentrations of lysine, isoleucine, leucine, phenylalanine, cysteine, and histidine than that from the Nili-Ravi breed [15]. 

A detailed fat and protein composition is critical for the processability and functionality of different dairy products. Therefore, this study aimed to generate an in-depth and comprehensive understanding of three buffalo breeds (Murrah, Nili-Ravi, and Mediterranean buffalo) and the macro- and micro-constituents of their milk, e.g., fat characterization (fat globule size, fatty acid profile, polar lipid content, and sterol content) and protein profiling (amino acid concentration and protein fraction content), which may provide a foundation of knowledge for Chinese dairy processors.

## 2. Materials and Methods

### 2.1. Materials

Fluorescent head-group-labeled phospholipid N-(Lissamine rhodamine B sulfonyl) dioleoylphosphatidylethanolamine (Rh-DOPE) was purchased from Avanti Polar Lipids (Alabaster, AL, USA). A standard mixture of 37 fatty acid methyl esters (FAME Mixture, C4 to C24) was obtained from Sigma-Aldrich (Shanghai, China). 

### 2.2. Buffalo Milk Samples 

Milk samples were collected from 10 Mediterranean, 10 Murrah, and 10 Nili-Ravi buffalos in January 2019 (Buffalo Research Institute Farm, Nanning, China). The milk samples were collected at 7:00 a.m. All buffalos were between the ages of 5 and 7 years, fed the same diets, and between 100 and 150 lactation days.

### 2.3. Gross Chemical Composition of Buffalo Milk

The milk samples were analyzed using milkoscan F120 (FOSS, Hillerød, Denmark) for their chemical composition (% fat, % protein, lactose, and % total solid).

### 2.4. Fat Globule Particle Size 

Fat globule size distributions were determined using a laser particle size analyzer (Microtrac Instruments, Montgomeryville, PA, USA) at room temperature. A total of 0.2 mL of buffer pH 7.0 (35 mM Ethylenediamine tetraacetic acid/sodium hydroxide and 1%, wt%) sodium dodecyl sulfate) was added to the 0.2 mL milk sample. The wavelength was set at 780 nm [16].

### 2.5. Fatty Acid Content

The milk samples were centrifuged at 5000× *g* for 20 min at 4 °C to separate the fat. The milk fatty acids were transformed into fatty acid methyl esters using 10 mg fat + 125 μL of 2 M KOH-CH3OH (KOH dissolved in methanol) and 700 μL of n-hexane and mixed for 2 min. Meanwhile, after one minute, sodium sulfate was added, mixed, and left to stand; then, the supernatant was collected and passed through a 0.22 µm filter. Fatty acid separation was carried out as described by Zou [17] using gas chromatography (GC, Agilent 7820 A system, Santa Clara, CA, USA) coupled with a capillary column (Trace TR-FAME, 60 m × 0.25 mm × 0.25 μm; Thermo Scientific, Waltham, MA, USA) and a hydrogen flame ionization detector.

### 2.6. Composition of Polar Lipids (PLs)

PLs were extracted and purified according to the methods of Avalli [18] modified by Wei [19]. PLs were identified and quantified using a Waters quadrupole time-of-flight (Q-TOF) mass spectrometry (MS) instrument. A PL analysis was carried out on a Waters ultra-performance liquid chromatography (UPLC) system equipped with a Xevo C2-S Q-TOF-MS spectrometer (Waters, Milford, MA, USA). A BEH-HILIC silica column (100 mm × 1 mm × 1.7 μm) was used to perform the chromatography separation. An LC-MS/MS data analysis was performed using Micromass MassLynx V4.1 system Waters software (Milford, MA, USA) [20].

### 2.7. Sterol Content

The sterol content in the milk fat was measured as described by Yao [21]. Briefly, the milk fat (100 mg) was saponified using KOH (2 mol/L) at 85 °C for 1 h. After being extracted with n-hexane, the unsaponifiable fraction was silylated, and the silicane derivatives were analyzed using gas chromatography–mass spectrometry (GC-MS) equipped with a DB-5 MS capillary column (30 m × 0.25 mm × 0.52 mm).

### 2.8. Amino Acid Content

The amino acid content of the buffalo milk samples was determined according to He [22]. In brief, the milk samples were hydrolyzed under a nitrogen atmosphere using 6 M hydrochloric acid at 110 °C for 24 h. The hydrolysates were filtered through a 0.22 μm syringe filter after mixing with NaOH (6 M). The amino acid content was determined using an amino acid analyzer (L-8900, Hitachi, Tokyo, Japan).

### 2.9. Protein Fraction

The protein fractions of the milk from the different buffalo breeds were measured according to Bobe [23] using RP-HPLC (Agilent 1260 Infinity, Santa Clara, CA, USA) equipped with a ZORBAX 300SB-C8 column (3.5 µm; 4.6 mm × 150 mm, Agilent, Santa Clara, CA, USA). The separation conditions were set as described by Bonfatti [24].

### 2.10. Statistical Analysis

The data were statistically analyzed using a one-way ANOVA in Statistix Software (Version 8.1, Institute, Inc., Cary, NC, USA). The results were considered statistically significant at *p* < 0.05.

## 3. Results

### 3.1. Chemical Composition

The chemical composition of the milk from the three buffalo breeds is presented in Table 1. The Mediterranean buffalo milk showed the highest percentage of total solids (TSs), solids, and fat (*p* < 0.05). However, the milk from Nili-Ravi was characterized by the lowest percentage of protein and solids not fat (SNF) (*p* < 0.05). Additionally, no significant difference (*p* ≥ 0.05) in the percentage of protein and SNF between the milk from the Murrah and Mediterranean buffalos was noted. Regarding lactose content, no significant differences (*p* ≥ 0.05) were recorded between the milk from the different buffalo breeds (Table 1). Moreover, no significant differences (*p* ≥ 0.05) in the chemical compositions (fat, lactose, and TSs) were found between the milk from the Murrah and Nili-Ravi breeds, except for the milk from Murrah, which contained significantly (*p* < 0.05) higher protein % and % SNF than the Nili-Ravi milk. In the same manner, Zhou [15] reported that milk from Murrah was characterized by a higher percentage of protein, fat, and TSs than milk from Nili-Ravi. In contrast, Sun [13] reported that milk from Nili-Ravi was characterized by higher % protein, fat, lactose, and TSs than milk from Mediterranean buffalo. The differences between the results of this report and the results of the studies by Sun [13] and Zhou [15] could be due to the impact of seasonal variations. In addition, diet composition has a significant impact on milk composition. Furthermore, the stage of lactation and lactation number have an effect on the chemical composition of buffalo milk [2]. In agreement with our results, Ahmad [8] reported similar fat (70 g/kg) and lactose (52.1 g/kg) contents of milk from the French Murrah buffalo breed. However, low protein (43.5 g/kg) and TSs (174.5 g/kg) were observed compared to our results. Moreover, milk from the Murrah buffalo breed showed the best performance of fat, total protein, and casein compared with that from the Bhadawari, Mehsana, and Surti buffalo breeds [25]. 

Compared with cow milk, the results of our study confirm that buffalo milk contains higher nutritive components [8].

### 3.2. Fat Characterization

#### 3.2.1. Fat Globule Size 

The fat globule size in the milk from the different buffalo breeds was measured using a laser particle size analyzer, and the results are given in Table 1. The diameter of the three buffalo breeds’ fat globules ranged from 3.88 to 5.67 µm. The milk from Murrah showed the biggest diameter of fat globules, while the smallest fat globule diameter was noted for the milk from the Mediterranean buffalo. Nevertheless, no significant difference (*p* ≥ 0.05) was noticed between Murrah and Nili-Ravi. In contrast with this finding, El-Zeini [6] stated that milk containing high fat usually has bigger fat globules.

The obtained significant differences in fat globule size between the breeds were most likely due to genetic variations, considering that the three breeds were fed with the same diet and had the same conditions of housing. In contrast with this result, Nguyen [26] and Ménard [7] reported a large fat globule size in milk from the Mediterranean breed (Cantal region, France), with an average diameter of 5.0 ± 0.4 μm compared to 3.88 ± 0.7 μm in this study, which could be attributed to feeding, housing conditions, and seasonal variations. In this context, it was reported that seasonal and lactational variations influenced the fat globule size [27]. Moreover, the relationship between the effects of breed and days in milking on fat globule size may be attributed to the relationship between breed and days in milking with fat yield [28]. The larger size of fat globules in Murrah milk is an important factor in the manufacture of cheese and fatty dairy products, and it affects the quality and physical properties of these products [29]. In general, the fat globule size in cow milk is smaller than that in buffalo milk due to the high fat content in buffalo milk [7]. 

#### 3.2.2. Fatty Acid Profile

The fatty acid contents of the milk fat from the three buffalo breeds are displayed in Table 2. The results show that palmitic acid (C16:0; 31.7 to 34.4%), oleic acid (C18:1; 24.59 to 27.22%), stearic acid (C18:0; 14.23 to 10.97%), and myristic acid (C14:0; 10.4 to 11.0%) were the main fatty acids in all buffalo milk samples. In agreement with this finding, Sun [13] reported that the above fatty acids were the main fatty acids in milk fat from the Murrah and Nili-Ravi buffalo breeds. The data in Table 2 reveal that the Mediterranean buffalo milk fat was characterized by the highest amount of caproic (C6:0), myristoleic (C14:1), pentadecanoic (C15:0), palmitic (C16:0), palmitoleic (C16:1), heptadecanoic (C17:0), heptadecenoic (C17:1), linoleic (C18:2C), γ-linolenic (C18:3N6), and dihomo-γ-linolenic acids (C20:3), while the Murrah buffalo milk fat exhibited the highest amount of stearic (C18:0), oleic (C18:1), linolelaidic (C18:2T), gondoic (C20:1), and α-linolenic acids (C18:3N3). In addition, the total unsaturated fatty acids were higher in the milk fat from the Murrah breed than in that from the Mediterranean and Nili-Ravi buffalo breeds. However, the milk fat from the Nili-Ravi buffalo breeds exhibited a higher amount of total saturated fatty acids than that from the Murrah and Mediterranean breeds (Table 2). Moreover, the total unsaturated fatty acids in the milk was higher in the milk fat from all buffalo breeds than in the milk fat from cow [7,21]. From a nutrition view, Murrah buffalo milk fat could be considered healthier due to it having a higher content of unsaturated fatty acids than the milk fat from the other two breeds. In contrast with these results, Sun [13] reported no significant difference in the individual fatty acid content in milk fat from the Murrah and Nili-Ravi buffalo breeds, except for the finding that caproic (C6:0) was significantly higher in milk fat from Murrah.

#### 3.2.3. Polar Lipid Profile 

The polar lipid profiles of the milk from the three buffalo breeds are listed in Table 3. The results indicate that phosphatidylethanolamine (PE), which ranged from 41.8 to 43.3%, was the main polar lipid fraction, followed by phosphatidylcholine (PC), which ranged from 29.8 to 30.3% (Table 3). Phosphatidylglycerol (PG) represented the lowest polar lipid fraction, ranging from 0.3 to 0.6%, in the lipids from the three buffalo breeds. It should be noted that no significant difference (*p* ≥ 0.05) was observed between the buffalo breeds in PC and PE%. Furthermore, the milk lipids from the Mediterranean breed exhibited the highest content of sphingomyelin (SM), while the highest content of phosphatidylinositol (PI) and PG was found in the Murrah breed and the Nili-Ravi breed, respectively. In agreement with these results, Ménard [7] revealed that PE, accounting for 29.4%, was a major fraction of the polar lipid classes in the milk fat from the Mediterranean breed. In contrast with these findings, PC was a major polar lipid fraction followed by PE in milk fat from Egyptian buffalo milk, which accounted for 35.56 and 17.37%, respectively [30]. These results demonstrate the effect of genetic and environmental conditions on milk fat characteristics. Moreover, Ménard et al. [7] reported no significant differences in polar lipid composition between milk fat from cow and buffalo.

This result is different from that of Holstein milk [31], which may be related to the breed and feeding method.

Studies have shown that PC is an important pathway of fatty acid transmission during brain development and growth and that it plays an important role in brain growth [32]. Moreover, PE has the effect of anti-apoptosis, which has been proven to induce the activation of mitogen-activated protein kinases (MAPKs) in PC12 cells, thus activating the MAPK cascade reaction to inhibit the apoptosis induced by serum deprivation [33]. Furthermore, PG is involved in the pathogenesis of cardiovascular diseases [34]. Buffalo milk is rich in PC and PE, which makes it suitable for the development of new functional lipid products.

#### 3.2.4. Sterol Content

The sterol content in the milk fat from the different buffalo breeds is presented in Table 4. Seven sterol fractions were identified in the milk fat from the three buffalo breeds: squalene, cholesterol, desmosterol, lathosterol, stigmasterol, beta-sitosterol, and lanosterol. Among these seven sterols, cholesterol, ranging from 235.2 to 289.2 mg/100 g fat, was the main sterol (Table 4). Additionally, beta-sitosterol was the lowest sterol presented in the milk fat of the three buffalo breeds. Furthermore, squalene was the main minor sterol in the buffalo milk fat. In contrast, lathosterol was the main minor sterol in the milk fat of cow [21]. The lipids of the Mediterranean buffalo milk exhibited significantly (*p* < 0.05) higher amounts of cholesterol and lanosterol than the lipids of the milk from the Murrah breed and the Nili-Ravi breed. This result is in parallel with the high fat content of the Mediterranean buffalo milk (Table 1). However, the lipids of the Nili-Ravi buffalo milk showed the highest (*p* < 0.05) amount of squalene, lathosterol, stigmasterol, and beta-sitosterol. In this context, the buffalo milk fat contained a lower cholesterol content than the milk fat of cow, goat, and camel [35]. However, the buffalo milk fat showed a higher content of lathosterol, desmosterol, dihyrdolanosterol, and lanosterol than the milk fat from cow, goat, and camel [35]. Interestingly, the cholesterol concentrations in this study were lower than the cholesterol content of bovine milk and Murrah buffalo milk [16,26]. 

### 3.3. Protein Characterization

#### 3.3.1. Amino Acid Profiles

The detailed amino acid contents of the milk protein from the three buffalo breeds are presented in Table 5. Milk is a rich source of essential amino acids and plays an important role in human nutrition. The data in Table 5 show that glutamic acid was the most abundant amino acid, with concentrations ranging from 18.83 to 19.69 g/100 g protein, followed by proline (9.64–9.78 g/100 g protein) and leucine (9.52–9.47 g/100 g protein). These results are in agreement those of with Sun [13] and Zhou [15], who reported that glutamic acid and proline were the predominant amino acids in Murrah and Nili-Ravi buffalo milk. As seen from the data given in Table 5, no significant (*p* ≥ 0.05) effect of buffalo breed on the amino acid contents was observed. Similar findings were obtained by Sun [13], who reported that there were no significant differences in the average values of individual amino acids between Murrah and Nili-Ravi buffalo milk. In contrast, Zhou [15] reported that Nili-Ravi buffalo milk was characterized by higher methionine than Murrah milk. Buffalo milk contains higher amino acid concentrations, which could be attributed to the higher protein content in buffalo milk.

#### 3.3.2. Protein Fractions

Milk protein consists of two major groups, namely, caseins and whey proteins. Casein contains subunits called alpha-s (αs), beta- (β), and Kapa (κ) caseins, while the major whey proteins are beta-lactoglobulin (β-Lg) and alpha-lactalbumin (α-La) [36]. The milk protein fractions of the three buffalo breeds’ milk were determined using RP-HPLC, and the results are shown in Table 6 as a percentage of total milk protein. It can be seen from the data presented in Table 6 that the milk protein fraction proportions significantly (*p* < 0.05) varied between the buffalo breeds. The Nili-Ravi buffalo milk protein was characterized by the highest proportion of casein fractions (αs-, β-, and κ-caseins), while the Murrah milk protein contained the highest amount of whey proteins (β-Lg and α-La). In contrast, Ren [14] reported that the protein fraction content was similar in milk from Murrah and Nili-Ravi, except for κ-casein, which was higher in the Nili-Ravi milk protein. In conclusion, the milk from all buffalo breeds contains a higher amount of casein fractions (αs- and κ-caseins), while the concentrations of the whey protein fractions are similar in cow and buffalo milk [2,4,37].

## 4. Conclusions

This work aimed to study the impact of buffalo breed (Murrah, Nili-Ravi, and Mediterranean) on the chemical composition, fat, and protein characteristics of buffalo milk. Mediterranean buffalo milk exhibited a higher content of protein, fat, and total solids, as well as a higher content of most fatty acids, than the milk from the other two breeds. In addition, buffalo breed had no impact on the lactose content in milk. However, the milk from Murrah showed the biggest diameter of fat globules. Moreover, total unsaturated fatty acids were higher in the milk fat of the Murrah breeds. Additionally, the milk fat of the Mediterranean breed contained the highest amount of SM, while the highest content of PI and PG was found in the Murrah breed and the Nili-Ravi breed, respectively. In addition, cholesterol concentrations were higher in the milk fat of the Mediterranean breed. Regarding milk protein characteristics, buffalo breed showed no significant effect on the amino acid profiles of the milk. Nevertheless, Nili-Ravi buffalo milk protein was rich in casein fractions, while Murrah milk protein was rich in whey proteins. The effects of breed variations on milk composition are widely known and represent a significant challenge for dairy processors. Further work is needed to identify a portfolio of significant composition–processing correlations that are linked to product quality. 

## Figures and Tables

**Table 1:** Chemical composition and fat globule size of milk from different buffalo breeds.

Buffalo Breeds	Protein%	Fat%	Lactose%	SNF%	TS%	Fat Globule Size (µm)
Nili-Ravi	4.38 ± 0.2 ^B^	6.84 ± 1.2 ^B^	5.27 ± 0.3 ^A^	9.70 ± 0.5 ^B^	17.29 ± 1.6 ^B^	6.53 ± 0.7 ^A^
Murrah	4.91 ± 0.2 ^A^	7.17 ± 1.7 ^B^	5.25 ± 0.2 ^A^	10.34 ± 0.2 ^A^	18.23 ± 1.8 ^B^	5.67 ± 0.6 ^A^
Mediterranean	5.08 ± 0.5 ^A^	8.98 ± 1.2 ^A^	4.98 ± 0.5 ^A^	10.17 ± 0.5 ^A^	20.18 ± 1.2 ^A^	3.88 ± 0.7 ^B^

Values are means of 10 samples ± SD. Different superscript letters in the same column mean significant differences (*p* < 0.05).

**Table 2:** Fatty acid (%) profile of milk from different buffalo breeds.

Fatty Acids	Buffalo Breeds		
	Nili-Ravi	Murrah	Mediterranean
C4:0	2.34 ± 0.2 ^A^	1.83 ± 0.2 ^A^	1.81 ± 0.3 ^A^
C6:0	1.42 ± 0.3 ^A^	0.92 ± 0.3 ^B^	1.56 ± 0.3 ^A^
C8:0	0.70 ± 0.1 ^AB^	0.64 ± 0.1 ^A^	0.77 ± 0.2 ^A^
C10:0	1.6 ± 0.2 ^A^	1.5 ± 0.2 ^A^	1.6 ± 0.3 ^A^
C11:0	0.04 ± 0.0 ^A^	0.05 ± 0.0 ^A^	0.03 ± 0.0 ^A^
C12:0	2.23 ± 0.1 ^A^	2.16 ± 0.2 ^A^	2.24 ± 0.3 ^A^
C13:0	0.07 ± 0.0 ^A^	0.08 ± 0.0 ^A^	0.06 ± 0.0 ^A^
C14:0	11.0 ± 1.1 ^A^	10.4 ± 1.3 ^A^	10.6 ± 0.8 ^A^
C14:1	0.78 ± 0.2 ^B^	0.79 ± 0.1 ^B^	1.14 ± 0.2 ^A^
C15:0	1.05 ± 0.1 ^B^	1.08 ± 0.1 ^AB^	1.19 ± 0.1 ^A^
C15:1	0.28 ± 0.0 ^B^	0.26 ± 0.0 ^B^	0.37 ± 0.1 ^A^
C16:0	33.0 ± 1.6 ^AB^	31.7 ± 1.8 ^B^	34.4 ± 1.5 ^A^
C16:1	2.10 ± 0.2 ^B^	2.07 ± 0.2 ^B^	2.52 ± 0.3 ^A^
C17:0	0.51 ± 0.1 ^B^	0.54 ± 0.0 ^B^	0.71 ± 0.0 ^A^
C17:1	0.23 ± 0.0 ^B^	0.26 ± 0.0 ^B^	0.35 ± 0.1 ^A^
C18:0	13.79 ± 0.9 ^A^	14.23 ± 1.0 ^A^	10.97 ± 0.8 ^B^
C18:1	24.59 ± 1.2 ^B^	27.22 ± 1.7 ^A^	25.76 ± 1.7 ^AB^
C18:2T	0.83 ± 0.1 ^A^	0.87 ± 0.1 ^A^	0.55 ± 0.2 ^B^
C18:2C	1.67 ± 0.1 ^B^	1.70 ± 0.1 ^B^	1.89 ± 0.2 ^A^
C18:3N6	0.14 ± 0.0 ^B^	0.14 ± 0.0 ^B^	0.19 ± 0.0 ^A^
C20:1	1.37 ± 0.1 ^B^	1.59 ± 0.1 ^A^	0.86 ± 0.2 ^C^
C18:3N3	0.26 ± 0.0 ^A^	0.28 ± 0.0 ^A^	0.21 ± 0.0 ^B^
C20:2	0.04 ± 0.0 ^A^	0.06 ± 0.0 ^A^	0.06 ± 0.0 ^A^
C20:3	0.07 ± 0.0 ^B^	0.07 ± 0.0 ^B^	0.08 ± 0.0 ^A^
C20:4	0.06 ± 0.0 ^A^	0.06 ± 0.0 ^A^	0.07 ± 0.0 ^A^
C22:0	0.1 ± 0.0 ^A^	0.1 ± 0.0 ^A^	0.09 ± 0.0 ^A^
C20:5	0.02 ± 0.0 ^A^	0.03 ± 0.0 ^A^	0.02 ± 0.0 ^A^
C22:2	0.04 ± 0.0 ^A^	0.05 ± 0.0 ^A^	0.06 ± 0.0 ^A^
C24:0	0.08 ± 0.0 ^A^	0.08 ± 0.0 ^A^	0.09 ± 0.0 ^A^
C24:1	0.02 ± 0.0 ^A^	0.03 ± 0.0 ^A^	0.03 ± 0.0 ^A^
C22:5N3	0.03 ± 0.0 ^B^	0.03 ± 0.0 ^B^	0.06 ± 0.0 ^A^
USFA	33.18 ± 1.4 ^B^	36.155 ± 1.8 ^A^	34.75 ± 2.1 ^AB^
SFA	67.49 ± 2.4 ^A^	64.561 ± 2.3 ^B^	65.94 ± 1.7 ^AB^

Values are means of 10 samples ± SD. Different superscript letters in the same row mean significant differences (*p* < 0.05). USFA, total unsaturated fatty acids; SFA, total saturated fatty acids.

**Table 3:** Polar lipid composition of milk from different breeds (% of polar lipids).

Buffalo Breeds	SM	PC	PE	PI	PG
Nili-Ravi	6.6 ± 0.5 ^B^	30.2 ± 1.3 ^A^	43.3 ± 2.9 ^A^	19.3 ± 1.5 ^B^	0.6 ± 0.2 ^A^
Murrah	6.9 ± 0.9 ^B^	29.8 ± 1.8 ^A^	41.2 ± 1.6 ^A^	21.9 ± 1.3 ^A^	0.3 ± 0.1 ^B^
Mediterranean	8.5 ± 1 ^A^	30.3 ± 1.9 ^A^	41.8 ± 2.5 ^A^	19.0 ± 1.5 ^B^	0.3 ± 0.1 ^B^

Values are means of 10 samples ± SD. Different superscript letters in the same column mean significant differences (*p* < 0.05). SM, sphingomyelin; PC, phosphatidylcholine; PE, phosphatidylethanolamine; PI, phosphatidylinositol; PG, phosphatidylglycerol.

**Table 4:** Sterol contents of milk of different breeds (mg/100 g).

Buffalo Breeds	Squalene	Cholesterol	Desmosterol	Lathosterol	Stigmasterol	Beta-Sitosterol	Lanosterol
Nili-Ravi	14.7 ± 2.8 ^A^	249.4 ± 8.7 ^B^	1.4 ± 0.8 ^A^	9.8 ± 1.1 ^A^	1.1 ± 0.4 ^A^	0.8 ± 0.3 ^A^	7.5 ± 0.9 ^A^
Murrah	10.2 ± 1.9 ^B^	235.2 ± 8.9 ^C^	1.5 ± 0.3 ^A^	7.1 ± 1.2 ^AB^	0.7 ± 0.18 ^B^	0.5 ± 0.1 ^B^	6.0 ± 1.3 ^B^
Mediterranean	11.1 ± 1.8 ^B^	289.2 ± 9.4 ^A^	1.3 ± 0.3 ^A^	6.3 ± 0.9 ^B^	1.0 ± 0.3 ^AB^	0.6 ± 0.1 ^B^	8.4 ± 0.9 ^A^

Values are means of 10 samples ± SD. Different superscript letters in the same column mean significant differences (*p* < 0.05).

**Table 5:** Amino acid (g/100 g protein) profile of milk from different buffalo breeds.

Amino Acids	Buffalo Breeds		
	Nili-Ravi	Murrah	Mediterranean
Aspartic acid	7.0 ± 0.7 ^A^	7.2 ± 0.1 ^A^	6.56 ± 0.3 ^A^
Methionine	2.51 ± 0.2 ^A^	2.58 ± 0.1 ^A^	2.5 ± 0.2 ^A^
Threonine	4.19 ± 0.3 ^A^	4.21 ± 0.31 ^A^	4.0 ± 0.1 ^A^
Serine	4.65 ± 0.3 ^A^	4.76 ± 0.2 ^A^	4.47 ± 0.2 ^A^
Valine	5.84 ± 0.5 ^A^	5.84 ± 0.6 ^A^	5.84 ± 0.4 ^A^
Phenylalanine	4.34 ± 0.2 ^A^	4.41 ± 0.1 ^A^	4.33 ± 0.3 ^A^
Leucine	9.52 ± 0.6 ^A^	9.51 ± 0.6 ^A^	9.47 ± 0.6 ^A^
Tyrosine	4.87 ± 0.3 ^A^	5.03 ± 0.1 ^A^	4.8 ± 0.3 ^A^
Lysine	7.61 ± 0.5 ^A^	7.67 ± 0.1 ^A^	7.35 ± 0.3 ^A^
Proline	9.74 ± 0.5 ^A^	9.64 ± 0.2 ^A^	9.78 ± 0.7 ^A^
Arginine	2.74 ± 0.2 ^A^	2.78 ± 0.1 ^A^	2.76 ± 0.2 ^A^
Histidine	2.51 ± 0.2 ^A^	2.51 ± 0.1 ^A^	2.43 ± 0.1 ^A^
Glycine	1.74 ± 0.1 ^A^	1.8 ± 0.1 ^A^	1.73 ± 0.1 ^A^
Alanine	2.97 ± 0.2 ^A^	2.99 ± 0.1 ^A^	2.82 ± 0.1 ^A^
Isoleucine	5.33 ± 0.3 ^A^	5.43 ± 0.1 ^A^	5.31 ± 0.3 ^A^
Glutamic acid	19.41 ± 0.6 ^A^	19.69 ± 0.6 ^A^	18.83 ± 0.4 ^A^

Values are means of 10 samples ± SD. Different superscript letters in the same row mean significant differences (*p* < 0.05).

**Table 6:** Protein fractions of milk from different buffalo breeds (as a percentage of total milk protein content).

Buffalo Breeds	κ-CN	αs-CN	β-CN	α-LA	β-LG
Nili-Ravi	13.4 ± 0.6 ^A^	36.5 ± 0.5 ^A^	35.5 ± 1.7 ^A^	4.98 ± 0.5 ^C^	8.97 ± 1.3 ^B^
Murrah	11.8 ± 0.7 ^C^	33.7 ± 1.7 ^B^	28.4 ± 1.9 ^B^	9.7 ± 1.5 ^A^	16.3 ± 1.1 ^A^
Mediterranean	12.6 ± 0.7 ^B^	34.2 ± 1.3 ^B^	30.4 ± 1.8 ^B^	7.7 ± 1.6 ^B^	15.1 ± 1.3 ^A^

Values are means of 10 samples ± SD. Different superscript letters in the same column mean significant differences (*p* < 0.05).

## Data Availability

Not applicable.

## References

[1] IDF (2021). Bulletin of the IDF N° 512/2021: The World Dairy Situation 2021.

[2] Abd El-Salam M.H., El-Shibiny S. (2011). A Comprehensive Review on the Composition and Properties of Buffalo Milk. Dairy Sci. Technol..

[3] Minervino A.H.H., Zava M., Vecchio D., Borghese A. (2020). *Bubalus bubalis*: A Short Story. Front. Vet. Sci..

[4] Garau V., Manis C., Scano P., Caboni P. (2021). Compositional Characteristics of Mediterranean Buffalo Milk and Whey. Dairy.

[5] Kapadiya D.B., Prajapati D.B., Jain A.K., Mehta B.M., Darji V.B., Aparnathi K.D. (2016). Comparison of Surti Goat Milk with Cow and Buffalo Milk for Gross Composition, Nitrogen Distribution, and Selected Minerals Content. Vet. World.

[6] El-Zeini H.M. (2006). Microstructure, rheological and geometrical properties of fat globules of milk from different animal species. Pol. J. Food Nutr. Sci..

[7] Ménard O., Ahmad S., Rousseau F., Briard-Bion V., Gaucheron F., Lopez C. (2010). Buffalo vs. Cow Milk Fat Globules: Size Distribution, Zeta-Potential, Compositions in Total Fatty Acids and in Polar Lipids from the Milk Fat Globule Membrane. Food Chem..

[8] Ahmad S., Gaucher I., Rousseau F., Beaucher E., Piot M., Grongnet J.F., Gaucheron F. (2008). Effects of Acidification on Physico-Chemical Characteristics of Buffalo Milk: A Comparison with Cow’s Milk. Food Chem..

[9] Gustavsson F., Buitenhuis A.J., Johansson M., Bertelsen H.P., Glantz M., Poulsen N.A., Lindmark Månsson H., Stålhammar H., Larsen L.B., Bendixen C. (2014). Effects of Breed and Casein Genetic Variants on Protein Profile in Milk from Swedish Red, Danish Holstein, and Danish Jersey Cows. J. Dairy Sci..

[10] Ma X., Liang S., Liang A., Rushdi H.E., Deng T. (2021). Evolutionary Analysis of OAT Gene Family in River and Swamp Buffalo: Potential Role of SLCO3A1 Gene in Milk Performance. Genes.

[11] Pineda P.S., Flores E.B., Herrera J.R.V., Low W.Y. (2021). Opportunities and Challenges for Improving the Productivity of Swamp Buffaloes in Southeastern Asia. Front. Genet..

[12] Borghese A., Moioli B. (2016). Management of Dairy Animals: Buffalo: Mediterranean Region. Encyclopedia of Dairy Sciences.

[13] Sun Q., Lv J., Liu L., Zhang S., Liang X., Lu J. (2014). Comparison of Milk Samples Collected from Some Buffalo Breeds and Crossbreeds in China. Dairy Sci. Technol..

[14] Ren D.X., Zou C.X., Lin B., Chen Y.L., Liang X.W., Liu J.X. (2015). A Comparison of Milk Protein, Amino Acid and Fatty Acid Profiles of River Buffalo and Their F1 and F2 Hybrids with Swamp Buffalo in China. Pak. J. Zool..

[15] Zhou L., Tang Q., Wasim Iqbal M., Xia Z., Huang F., Li L., Liang M., Lin B., Qin G., Zou C. (2018). A Comparison of Milk Protein, Fat, Lactose, Total Solids and Amino Acid Profiles of Three Different Buffalo Breeds in Guangxi, China. Ital. J. Anim. Sci..

[16] Wei W., Yang J., Yang D., Wang X., Yang Z., Jin Q., Wang M., Lai J., Wang X. (2019). Phospholipid Composition and Fat Globule Structure I: Comparison of Human Milk Fat from Different Gestational Ages, Lactation Stages, and Infant Formulas. J. Agric. Food Chem..

[17] Zou X., Huang J., Jin Q., Guo Z., Liu Y., Cheong L., Xu X., Wang X. (2013). Lipid Composition Analysis of Milk Fats from Different Mammalian Species: Potential for Use as Human Milk Fat Substitutes. J. Agric. Food Chem..

[18] Avalli A., Contarini G. (2005). Determination of Phospholipids in Dairy Products by SPE/HPLC/ELSD. J. Chromatogr. A.

[19] Wei W., Li D., Jiang C., Zhang X., Zhang X., Jin Q., Zhang X., Wang X. (2022). Phospholipid Composition and Fat Globule Structure II: Comparison of Mammalian Milk from Five Different Species. Food Chem..

[20] Ali A.H., Zou X., Lu J., Abed S.M., Yao Y., Tao G., Jin Q., Wang X. (2017). Identification of Phospholipids Classes and Molecular Species in Different Types of Egg Yolk by Using UPLC-Q-TOF-MS. Food Chem..

[21] Yao Y., Zhao G., Xiang J., Zou X., Jin Q., Wang X. (2016). Lipid Composition and Structural Characteristics of Bovine, Caprine and Human Milk Fat Globules. Int. Dairy J..

[22] He J., Xiao Y., Orgoldol K., Ming L., Yi L., Ji R. (2019). Effects of Geographic Region on the Composition of Bactrian Camel Milk in Mongolia. Animals.

[23] Bobe G., Beitz D.C., Freeman A.E., Lindberg G.L. (1998). Separation and Quantification of Bovine Milk Proteins by Reversed-Phase High-Performance Liquid Chromatography. J. Agric. Food Chem..

[24] Bonfatti V., Grigoletto L., Cecchinato A., Gallo L., Carnier P. (2008). Validation of a New Reversed-Phase High-Performance Liquid Chromatography Method for Separation and Quantification of Bovine Milk Protein Genetic Variants. J. Chromatogr. A.

[25] Misra S., Sharma A., Bhattacharya T., Kumar P., Saha Roy S. (2008). Association of Breed and Polymorphism of As1-and As2-Casein Genes with Milk Quality and Daily Milk and Constituent Yield Traits of Buffaloes (*Bubalus bubalis*). Buffalo Bull..

[26] Nguyen H.T.H., Ong L., Beaucher E., Madec M.-N., Kentish S.E., Gras S.L., Lopez C. (2015). Buffalo Milk Fat Globules and Their Biological Membrane: In Situ Structural Investigations. Food Res. Int..

[27] Schafberg R., Schmidt R., Thiele M., Swalve H.H. (2007). Fat Globule Size Distribution in Milk of a German Buffalo Herd. Ital. J. Anim. Sci..

[28] Fleming A., Schenkel F.S., Chen J., Malchiodi F., Ali R.A., Mallard B., Sargolzaei M., Corredig M., Miglior F. (2017). Variation in Fat Globule Size in Bovine Milk and Its Prediction Using Mid-Infrared Spectroscopy. J. Dairy Sci..

[29] Michalski M.-C., Camier B., Briard V., Leconte N., Gassi J.-Y., Goudédranche H., Michel F., Fauquant J. (2004). The Size of Native Milk Fat Globules Affects Physico-Chemical and Functional Properties of Emmental Cheese. Lait.

[30] Ali A.H., Wei W., Abed S.M., Korma S.A., Mousa A.H., Hassan H.M., Jin Q., Wang X. (2018). Impact of Technological Processes on Buffalo and Bovine Milk Fat Crystallization Behavior and Milk Fat Globule Membrane Phospholipids Profile. LWT.

[31] Jiang C., Zhang X., Yu J., Yuan T., Zhao P., Tao G., Wei W., Wang X. (2022). Comprehensive Lipidomic Analysis of Milk Polar Lipids Using Ultraperformance Supercritical Fluid Chromatography-Mass Spectrometry. Food Chem..

[32] Nguyen L.N., Ma D., Shui G., Wong P., Cazenave-Gassiot A., Zhang X., Wenk M.R., Goh E.L.K., Silver D.L. (2014). Mfsd2a Is a Transporter for the Essential Omega-3 Fatty Acid Docosahexaenoic Acid. Nature.

[33] Nishina A., Kimura H., Sekiguchi A., Fukumoto R., Nakajima S., Furukawa S. (2006). Lysophosphatidylethanolamine in Grifola Frondosa as a Neurotrophic Activator via Activation of MAPK. J. Lipid. Res..

[34] Tan S.T., Ramesh T., Toh X.R., Nguyen L.N. (2020). Emerging Roles of Lysophospholipids in Health and Disease. Prog. Lipid. Res..

[35] Dhankhar J., Sharma R., Indumathi K.P. (2020). A Comparative Study of Sterols in Milk Fat of Different Indian Dairy Animals Based on Chemometric Analysis. J. Food Meas. Charact..

[36] Xiong Y.L. (2009). Dairy Proteins. Ingredients in Meat Products.

[37] Addeo F., Alloisio V., Chianese L., Alloisio V. (2007). Tradition and Innovation in the Water Buffalo Dairy Products. Ital. J. Anim. Sci..

